# Vaccines and Antiviral Therapies for Mpox Virus in Pregnant and Breastfeeding Women: Efficacy and Maternal–Child Outcomes

**DOI:** 10.3390/v17040456

**Published:** 2025-03-22

**Authors:** Maryum Imran, Myra Sohail, Javeria Kamran, Syeda Qaima Abbas, Khadija Azeem, Emmanuel Korir

**Affiliations:** 1 Department of Medicine, Dow University of Health Sciences, Karachi 74200, Pakistan; javeriakamran32@gmail.com; 2 Department of Medicine, Ziauddin Medical College, Karachi 75000, Pakistan; sohailmyra40@gmail.com; 3 Department of Medicine, Jinnah Sindh Medical University, Karachi 75510, Pakistan; qaima48@gmail.com; 4 Department of Medicine, Bahria University Health Sciences, Karachi 75500, Pakistan; azeemkhadija.ka@gmail.com; 5 Department of Medicine, University of Nairobi, Nairobi P.O. Box 30197-00100, Kenya; emmanuelqkorir@gmail.com

**Keywords:** monkeypox, mpox, pregnancy, lactation, breastfeeding, vertical transmission, vaccine, antiviral

## Abstract

Mpox (formerly known as monkeypox), the major public health concern of 2022, has elicited much attention globally. In addition to the usual symptoms observed in smallpox virus infections, infected mothers were found to hold a possible risk of transmission to newborns during delivery. This review aimed to summarize recent clinical trials that involved antiviral therapy, vaccines, immunoglobulin therapy, and other pharmacological interventions specifically for treating infected pregnant women. A comprehensive search was performed using databases such as PubMed, Google Scholar, and Medline to find appropriate disease management strategies. Amongst the vaccines and antivirals being used for treatment, vaccines such as Modified Vaccinia Ankara (MVA/MVA-BN) and Lister clone 16-medium pocket size-8 (LC16m8), while prophylactically effective, have been deemed unsafe for pregnant and lactating females. Antivirals like Tecovirimat, on the other hand, are considered to be a better alternative, but they are not without risks that may outweigh the potential benefits. Additionally, efforts to reduce maternal and fetal complications include administering the MVA-BN vaccine and awareness campaigns regarding herd immunity. Therefore, necessary precautions, prophylactic vaccinations in high-risk outbreak regions, and symptomatic treatment in pregnant and lactating females currently appear to be more feasible approaches against the mpox virus.

## 1. Introduction

Mpox (formerly monkeypox), an endemic disease in West and Central Africa, was first documented in the Democratic Republic of the Congo (DRC) in 1970 [1]. It emerged as a global public health concern in 2022 following the initial reported case in the UK [2]. Since 1 January 2022, the US Centers for Disease Control and Prevention (CDC) have reported a total of more than 102,000 cases worldwide, with more than 220 reported deaths [3]. However, by January 2025, the number of cases worldwide had steadily declined to nearly 3700 [4]. The virus, belonging to the orthopoxvirus genus, has two genetic clades: the Central African Clade (CA, Clade I) and the West African Clade (WA, Clade II), with the latter being more widespread [5]. The clinical symptoms of the disease bear a significant resemblance to those of the smallpox virus. This includes a notable skin rash following a similar progression, beginning with a plaque that evolves into papules, blisters, pustules, and eventually a scab. The skin lesions first appear on the face, then spread to other body areas, finally affecting the hands and feet [6]. While the prodromal period, beginning prior to the onset of rash, resembles that of the smallpox virus, albeit to a milder extent (in terms of fever, chills, malaise, body ache, and headache), the presentation of lymphadenopathy distinguishes the mpox virus from its counterpart [7]. Transmission was shown to occur through respiratory secretions [7], but recent cases, especially among homosexual men, also suggest sexual contact as a significant route [8]. Given that lesions can appear in the genital area, there is a risk of vertical transmission among pregnant women, particularly during childbirth [9]. Studies indicate that infected mothers can transmit the virus to newborns during delivery if they display distinct genital lesions [10,11]. However, if a mother is only seropositive without presenting any symptoms, significant risks to the neonate are not reported. Despite this, immediately after birth, protocols suggest implementing preventive measures. These include isolating the mother, using face masks, restricting breast milk, and testing the newborn for the virus [12].

Since the declaration of the virus as a Public Health Emergency of International Concern (PHEIC) by the WHO [13], ongoing investigations into clinical trials have focused on effective therapies. Recent trials using antiviral therapy in animal models showed promise in delaying disease progression and alleviating symptoms [14]. However, data on these interventions’ benefits for pregnant and lactating women are limited. This review aimed to summarize recent clinical trials that involved antiviral therapy, vaccines, immunoglobulin therapy, and other pharmacological interventions for infected pregnant women, which involved evaluating their efficacy, safety profiles, and potential teratogenic risks. The goal was to develop evidence-based guidelines for managing congenital mpox virus and post-delivery infections.

## 2. Methodology

This literature review compiled and analyzed the data on the efficacy and side effects of vaccines and antiviral therapies on pregnant or lactating women with mpox. A comprehensive search was performed, and relevant information was extracted from articles published in English on PubMed, Google Scholar, and Medline from the years 2018 to 2024. The keywords used in the search strategy were “mpox”, “monkeypox”, “monkeypox virus”, “pregnancy”, “pregnant”, “lactating”, “transmission”, “vaccine”, “antiviral”, “tecovirimat”, “cidofovir”, and “brincidofovir”.

## 3. Pathophysiology

Mpox is a self-limiting illness, the course of which is influenced by several factors, including the viral strain, the immunocompetence of the host, and the presence of complications [15]. It can be transmitted via respiratory droplets or contact with skin lesions of infected individuals [16]. Contact with a diseased animal and its bodily secretions can also transmit the virus [17]. Some studies propose that sexual transmission of the virus might be possible. The presence of mpox in seminal fluid and the elevated incidence of primary genital and anal mucosal lesions following unprotected sexual intercourse during the 2022 epidemic adds support to this claim [17]. The virus replicates at the site of infection before spreading to adjacent lymph nodes [18]. Subsequently, the mpox virus infiltrates the circulation, initiating a primary viremia that disseminates throughout the hematopoietic system. The time of primary viremia aligns with the incubation phase of the infection, which typically lasts 1–2 weeks [16]. During this stage, people infected with the virus are often asymptomatic and do not have skin lesions. After the latent phase, further replication of the mpox virus leads to secondary viremia [16], resulting in the display of prodromal symptoms, such as fever and chills, headache, myalgia, and lymphadenopathy, for up to three days [15]. Subsequently, the virus inoculates the skin and mucus membranes [16], leading to the development of rashes on the head and face, progressively disseminating over the body. The rash progresses from papules to vesicles and pustules to crusts that result in scarring. The rash lasts 2–4 weeks [15]. Vulnerable groups in the population, such as children, the elderly, and immunocompromised individuals, are more likely to suffer from complications, including hemorrhage, necrosis, multi-organ inflammation, and septicemia [15].

Though there is scarce research on the effect of mpox on pregnancy and fetal outcomes [16], studies determined that the virus can be transmitted from an infected mother to her fetus transplacentally or through direct contact during delivery [19]. A study of cases from the Democratic Republic of the Congo (DRC) found that women infected with the mpox virus may have a wide range of outcomes, from no effect on the child in mild cases to early miscarriages, stillbirth, and preterm birth in moderate and severe cases [20,21]. Some children who were born to infected mothers displayed signs of mpox infection, including a rash, hepatomegaly, hydrops fetalis [16], and high viral loads, whereas some remained completely unaffected [19]. A recent study identified four distinct mechanisms through which mpox infection may lead to vertical transmission [20,21]. One of these methods is the maternal–fetal transfer of the mpox virus after maternal viremia, wherein the virus accesses the placenta via the uterine arterial blood supply. In this instance, there will be maternal viremia and Hofbauer cells in the chorionic villi of the placenta [18]. Studies speculate that the virus enters the intervillous space from the mother’s uterine spiral arteries and attaches itself to trophoblast cells. It then infects syncytiotrophoblasts, cytotrophoblasts, fetal endothelial cells in the floating or anchored villi, and fetal blood cells. Additionally, the mpox virus might invade the decidua and chorionic membranes after ascending from vaginal lesions through cervical and uterine tissue. Mpox might also cross the maternal–placental–fetal barrier by fusing with trophoblasts, a process in which viral capsid proteins adhere to target cell surface receptors and change configuration, allowing viral DNA to be internalized via fusion with the syncytiotrophoblast membrane or transcytosis. Internalized viruses can cause damage to the host cells directly or indirectly by stimulating inflammation and an immune reaction [16].

## 4. Vaccines for Mpox: Efficacy and Maternal–Fetal Outcomes

There are two types of vaccines administered to people at high risk of infection. These are Modified Vaccinia Ankara (MVA/MVA-BN) and Lister clone 16-medium pocket size-8 (LC16m8). MVA-BN is a live, attenuated vaccine that does not replicate inside the human body. The vaccine binds T cells to antigen-presenting cells and prompts the cytotoxic T cells to kill virus-infected cells. It also activates B lymphocytes that form antibodies against the virus [22]. A recent study found that the risk of disease was reduced by 20% when used as a post-exposure vaccine [23] and by 62–85% when used as a primary prevention vaccine [24]. Various studies have reported an efficacy of 76% with a single dose and an efficacy of 82% with two doses [24]. The overall efficacy of this vaccine is 80% [24]. It is tolerated well. Common adverse effects include hypersensitivity, fatigue, nausea, and chills. LC16m8 is a live, attenuated vaccine with minimal ability to replicate. The mechanism of action of this vaccine is similar to that of MVA-BN. In addition to producing antibodies, it also produces a strong cellular response [22]. It was originally developed to prevent smallpox in the 1970s, but in 2022, Japan’s Pharmaceuticals and Medical Devices Agency (PMDA) concluded that it showed cross-reactivity with the mpox virus. The efficacy of this vaccine in humans has not been established yet. This vaccine is contraindicated in immunocompromised patients and in pregnancy [25].

There are limited data on the use of mpox vaccines in pregnancy, but it is highly recommended in high-risk populations to prevent the development of the disease in the mother and the child. It has been noted that smallpox vaccines have cross-reactivity against mpox and, as such, may be used to control its spread. Data collected from five small studies show that there was a small risk of congenital defects, stillbirths, and miscarriages when the vaccine was administered to the mother in the first trimester [26]. However, another study found no association between congenital defects and vaccine administration in the first trimester of pregnancy [27]. The vaccine has so far only been administered to 300 pregnant women, and no adverse effects were reported [28]. There was also no association found between vaccine administration in pregnancy and abortion or stillbirth. MVA-BN is recommended in pregnancy if the mother is at risk of infection. As it is a non-replicating vaccine, it is safer to use during pregnancy and lactation than the LC16m8 vaccine, which has some ability to replicate and may cross the placenta or be transmitted to the newborn via breast milk from an infected mother. However, there is no such evidence that might prove this. Nonetheless, the LC16m8 vaccine is contraindicated in pregnancy [25]. There are limited data available on the long-term effects of the mpox vaccine, as the vaccine is relatively new. Follow-up studies on vaccinated pregnant women might help determine the side effects of mpox vaccines down the line. A clinical trial that will evaluate the efficacy of MVA-BN in pregnant and lactating women, as well as in infants, will be conducted in early 2025 in the Congo. It will be a randomized controlled trial with a 12-month follow-up period [29].

## 5. Antiviral Therapies for Mpox: Efficacy and Maternal–Fetal Outcomes

Although data on the administration of mpox antivirals in pregnancy are limited, research that involved antiviral treatment for hepatitis B during pregnancy established a strong foundation for the use of antivirals in this context. Antivirals for hepatitis B demonstrated overall efficacy and safety regarding maternal and infant outcomes [30]. With this knowledge in mind, the impact of mpox antivirals on pregnancy was explored in recent years.

Tecovirimat is an antiviral agent that is especially effective against orthopoxviruses, such as smallpox, cowpox, and mpox [31]. By inhibiting the function of an envelope-wrapping protein, specifically the viral protein p37, the drug prevents cell-to-cell virus transmission by immobilizing the virus within the infected cell [31]. Pregnant individuals diagnosed with mpox and treated with tecovirimat may exhibit potential benefits. A case report of a pregnant female who was administered tecovirimat for mpox resulted in the delivery of a healthy baby who tested positive for IgG antibodies against orthopoxvirus but did not show any clinical findings indicative of an active infection [32]. While some studies suggest that there are no harmful short-term effects of tecovirimat on the mother and child [32], a recent trial in the Democratic Republic of Congo showed tecovirimat to have less than satisfactory results [33]. The disappointing result of this study involving 600 participants was attributed to there being no observable difference in terms of lesion clearing when the experimental group was compared with the placebo group [33]. Interestingly, tecovirimat was shown to be ineffective against certain mpox strains that contained mutations in specific enzymes involved in the formation of the virus’s outer envelope [34]. This resistance shown by mpox variants questions the effectiveness of tecovirimat.

Other antiviral agents that have been used to treat mpox are cidofovir and its pro-drug brincidofovir, which are DNA polymerase inhibitors and, therefore, inhibitors of DNA replication in orthopoxviruses [35]. According to a study that reviewed the pharmacokinetic and pharmacodynamic characteristics of these drugs, while cidofovir and brincidofovir were active against other dsDNA viruses, tecovirimat was found to be specific for orthopoxviruses [35]. As for their use in pregnancy, with tecovirimat, there were no fetal toxicities observed in animal studies. Brincidofovir was shown to have embryotoxicity in rat and rabbit models and, therefore, is suspected to cause fetal harm in humans [35]. Cidofovir is also contraindicated in pregnancy due to potential embryotoxic consequences [33]. Ultimately, there are insufficient data regarding the efficacy of cidofovir in human subjects infected with mpox, necessitating further research in this domain [35].

## 6. Comparative Analysis of Vaccine vs. Antiviral Therapies

Vaccines generally demonstrate a higher protective efficacy against mpox and may prevent hospitalization. The vaccine effectiveness for a single dose of MVA-BN (JYNNEOS) was 76% (95% CI 64–88%) across 12 studies and 82% (95% CI 72–92%) from 6 studies for two doses of MVA-BN in a systematic review carried out by Pischel et al. [24]. Additionally, MVA-BN reduced hospitalization with a vaccine effectiveness of 67% (95% CI 55–78%) [24]. In contrast, antiviral therapies, such as tecovirimat, brincidofovir, and cidofovir, are indicated for the post-exposure treatment of mpox. Given the limited clinical data on the efficacy of tecovirimat, clinical trials are currently underway to evaluate its therapeutic potential. Vaccinia Immune Globulin Intravenous (VIGIV) is indicated for the treatment of severe or complicated cases of mpox disease, requiring a thorough evaluation of the potential risks and benefits for each patient [12].

Vaccines are relatively safer for use in pregnant and lactating women compared with antiviral therapies. As a non-replicating vaccine, MVA-BN has lower side effect rates than replicating vaccines, such as ACAM200. MVA-BN is approved by the Food and Drug Administration (FDA) to prevent smallpox and mpox diseases. Currently, the data on the use of MVA-BN on pregnant and lactating women are limited. However, animal studies that involved rats and rabbits demonstrated no evidence of harm to the fetus [36]. Conversely, evidence from animal studies suggests that antiviral medications, including cidofovir and brincidofovir, may have teratogenic properties. In one animal study, cidofovir was associated with fetal growth restriction and musculoskeletal abnormalities [12]. In animal reproduction studies, brincidofovir was also associated with embryotoxicity and structural malformations [37]. Given these findings, administering these medications during the first trimester of pregnancy is not recommended. Furthermore, no data are available for assessing the risks in lactating women. Tecovirimat has not been studied in pregnant and lactating individuals. However, no embryo–fetal abnormalities have been recorded in reproductive animal studies. VIGIV is classified as pregnancy category C, indicating a potential risk to the developing fetus [38].

Antiviral medications present distinct adverse effects, which highlights critical consideration for their use among pregnant and lactating women. Tecovirimat is well-tolerated, as evidenced by a randomized, double-blind, multicenter phase III study (NCT02474589) involving healthy adult volunteers [39]. The study, which assessed tecovirimat administered orally at 600 mg twice daily for 14 days, reported no patterns of troubling treatment-emergent adverse events (TEAEs). TEAEs occurred in 37.3% out of 359 tecovirimat recipients and 33.3% out of 90 placebo recipients. Common adverse effects were headache (12 vs. 8% of subjects), nausea (5 vs. 4%), abdominal pain (2 vs. 1%), and vomiting (2 vs. 0%) [39]. Brincidofovir has been associated with hepatotoxicity, raising concerns about its use in pregnancy. Cidofovir has also been associated with nephrotoxicity, limiting its use to severe cases of mpox disease. VIGIV is generally safe but may cause infusion-related reactions, such as fever and chills [40].

Vaccines are the preferred option for preventing mpox infection. This intervention is indicated for individuals deemed at high risk of contracting mpox. The Centers for Disease Control and Prevention (CDC) guidelines state that MVA-BN can be safely administered to eligible pregnant or breastfeeding women. A study conducted by Ververs et al. shifted the focus to the WHO guidelines regarding mpox virus vaccination and breastfeeding women. The WHO 2024 guidelines mentioned the use of MVA-BN vaccination in breastfeeding women but raised valid concerns due to the lack of data regarding the recommendation and fear of repetition of the Ebola vaccine’s hesitancy in breastfeeding women. When the Ebola vaccine was first introduced in the DR Congo, many women chose to give up breastfeeding after getting the vaccine, believing that the vaccine would be harmful to their child, whereas others chose to forego the vaccine in favor of continuing to breastfeed. In a resource-poor country, it was a difficult choice to make. Although the concerns were alleviated after four years of thorough research and the vaccine was considered safe, it became an important criterion for future vaccinations [41]. A more recent article by Hombach et al. clarifies that the use of MVA-BN is safe as primary or post-exposure prevention for breastfeeding women at high risk, i.e., those with contact history, healthcare workers, etc. It highly supported breastfeeding by women in endemic areas and discouraged mothers from choosing between disease prevention via vaccination and breastfeeding; both can be accomplished simultaneously [42]. Vaccination is recommended before or promptly after exposure to ensure optimal efficacy in preventing or attenuating the disease severity. ACAM200 and minimally replicating vaccines, such as LC16m8, should be avoided in pregnant and lactating women. Under the expanded access Investigational New Drug (EA-IND) protocol, the CDC allowed for the use of tecovirimat for the primary or early empiric treatment of mpox [43]. Cidofovir, brincidofovir, and VGIV are considered alternative antiviral medications to treat mpox disease.

## 7. Current Knowledge Gap and Future Directions

Mpox, a previously contained disease endemic in some cities of Africa, became a global threat starting in May 2022 and an international concern by 12 August 2024, as declared by the World Health Organization (WHO) [44]. Currently, extensive work has been in progress to achieve proper management guidelines to control the spread of the virus and the severity of the symptoms in those already infected. Prior literature suggests that although vaccinations are available for most patients, these are not suitable for immunocompromised patients, including pregnant females and children. The safety of the community relies on undertaking preventive measures, especially for high-risk or exposed individuals. According to WHO guidelines, isolation and the use of personal protective equipment (PPE) by healthcare workers should be observed. Moreover, precautions regarding contact and airborne spread are also necessary. Infected individuals should use separate utilities, and the contact surfaces of fomites should be sterilized by a disinfectant. Although herd immunity for the mpox virus is not a requirement, it has been noticed that post-exposure prophylaxis vaccinations have been effective in controlling the spread, as well as the symptomatic nature of the disease, especially within 3–4 days until 14 days [45]. Therefore, mpox vaccination should be recommended to high-risk individuals, including men who have sex with men, sex workers, and healthcare workers, to limit the virus spread [40].

Due to the progressive increase in the incidence of disease cases, further research on the effect of this virus on susceptible members of our society is essential. Since the COVID-19 pandemic, the general public understands the purpose of vaccination and is more open to getting vaccinated. Required vaccinations should be made available in countries suffering from the virus outbreak, especially low-resource countries, and policies advocating for controlling the virus spread should be initiated [45]. Furthermore, safer, innovative medications and vaccinations suitable for pregnant females and breastfeeding mothers call for necessary resource investments. Expecting mothers are a vulnerable target, and additional research is required to prevent the rising numbers of maternal and fetal mortality due to the virus. At the community level, awareness about adverse symptoms of the virus through available media platforms will allow for early screening and testing. Combined combative action taken by the authorities and the general public will sufficiently eradicate the spread of this virus.

## 8. Conclusions

The mpox virus is becoming an imminent concern worldwide and requires strategic and advanced therapeutic techniques. While curative plans for the associated viral disease are being sought, current guidelines also prioritize raising public awareness and promoting early screening. Efforts to prevent maternal and fetal complications include early screening; administering the MVA-BN vaccine, a non-replicating vaccine with no reported fetal toxicity effects in animal studies; and raising awareness about herd immunity to protect vulnerable people in the community from those that are infected. The adverse effects of antivirals outweigh the benefits in pregnant and lactating females. Due to the unpredicted insurgence of this virus on a large scale, there is not much research favoring antivirals like Brincidofovir and Cidofovir. As observed in some animal studies, Tecovirimat has the potential to be effective in pregnant females with a reduced teratogenicity risk. Recent findings, however, did not demonstrate Tecovirimat to be particularly effective against mpox variants and it warrants further clinical trials. Vaccines, on the other hand, are considered to be a safer approach in these perilous maternal and neonatal conditions, even though there is a need for more clinical data supporting the effectiveness and safety of both types of treatments. Necessary individualized precautions, prophylactic vaccinations in high-risk outbreak regions, and symptomatic treatment in pregnant and lactating females are becoming the standard steps against the mpox virus. As learnt from the previous COVID-19 pandemic, containment and disease isolation plans should be implemented to ensure the safety of immunocompromised individuals in society.

## Abbreviations

The following abbreviations are used in this manuscript:

DRC	Democratic Republic of Congo
CDC	Centers for Disease Control and Prevention
CA	Central African Clade
WA	West African Clade
PHEIC	Public Health Emergency of International Concern
WHO	World Health Organization
Mpox	Monkeypox
MVA/MVA-BN	Modified Vaccinia Ankara
LC16m8	Lister clone 16-medium pocket size-8
PMDA	Pharmaceuticals and Medical Devices Agency
VIGIV	Vaccinia Immune Globulin Intravenous
FDA	Food and Drug Administration
TEAEs	Treatment-emergent adverse events
EA-IND	Expanded Access Investigational New Drug
PPE	Personal protective equipment
